# Supplementary material for: SCEPTRE improves calibration and sensitivity in single-cell CRISPR screen analysis
Source: Genome Biol. 2021 Dec 20;22:344. doi: 10.1186/s13059-021-02545-2 (PMC8686614; doi:10.1186/s13059-021-02545-2)
Supplement: Supplementary file 1 — Additional file 1 Supplementary figures and tables [file 13059_2021_2545_MOESM1_ESM.pdf]

Supplementary figures and tables

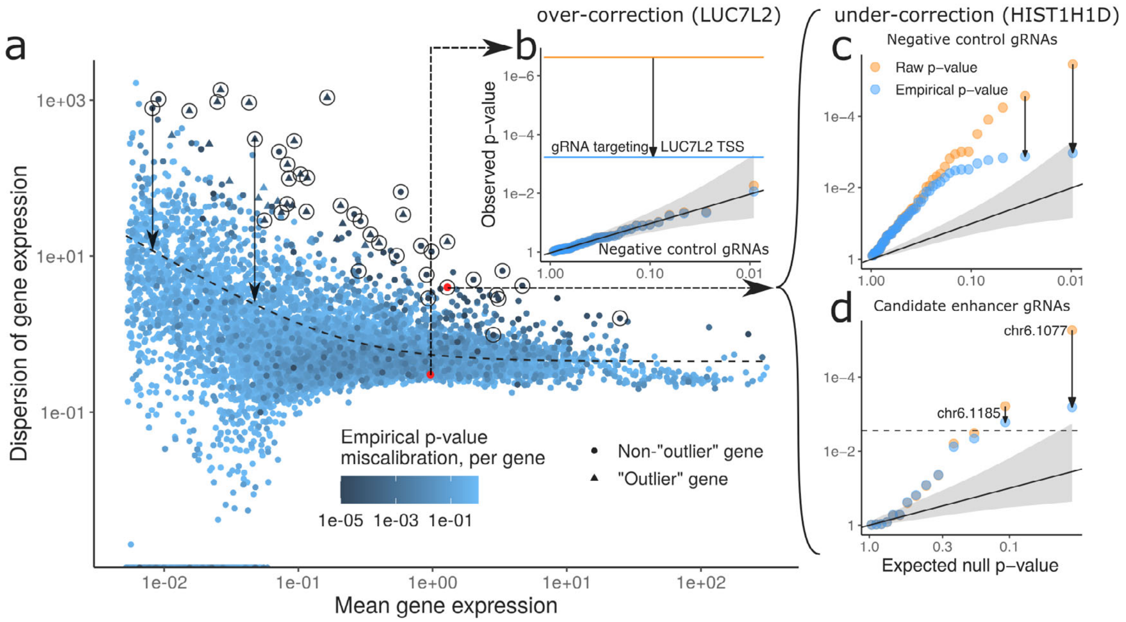

Figure S1: **Gasperini et al.'s empirical correction is insufficient to correct for miscalibration**. **a,** Dispersion estimation procedure employed leads to miscalibration for high-dispersion genes, which the empirical correction does not adequately correct for, as measured by KS test applied to empirical p-values per gene (point colors). **b,** Raw p-values already well-calibrated for *LUC7L2* gene, so empirical correction unnecessarily shrinks the significance of the association with TSS-targeting gRNA, depicted by horizontal lines, by three orders of magnitude. **c,** Empirical correction not strong enough for *HIST1H1D*, which is among circled genes in panel a, which have an NTC-based miscalibration p-value smaller than the Bonferroni threshold. **d**, Under-correction leads to two potential false discoveries for *HIST1H1D*. Dashed horizontal line represents the multiple testing threshold.

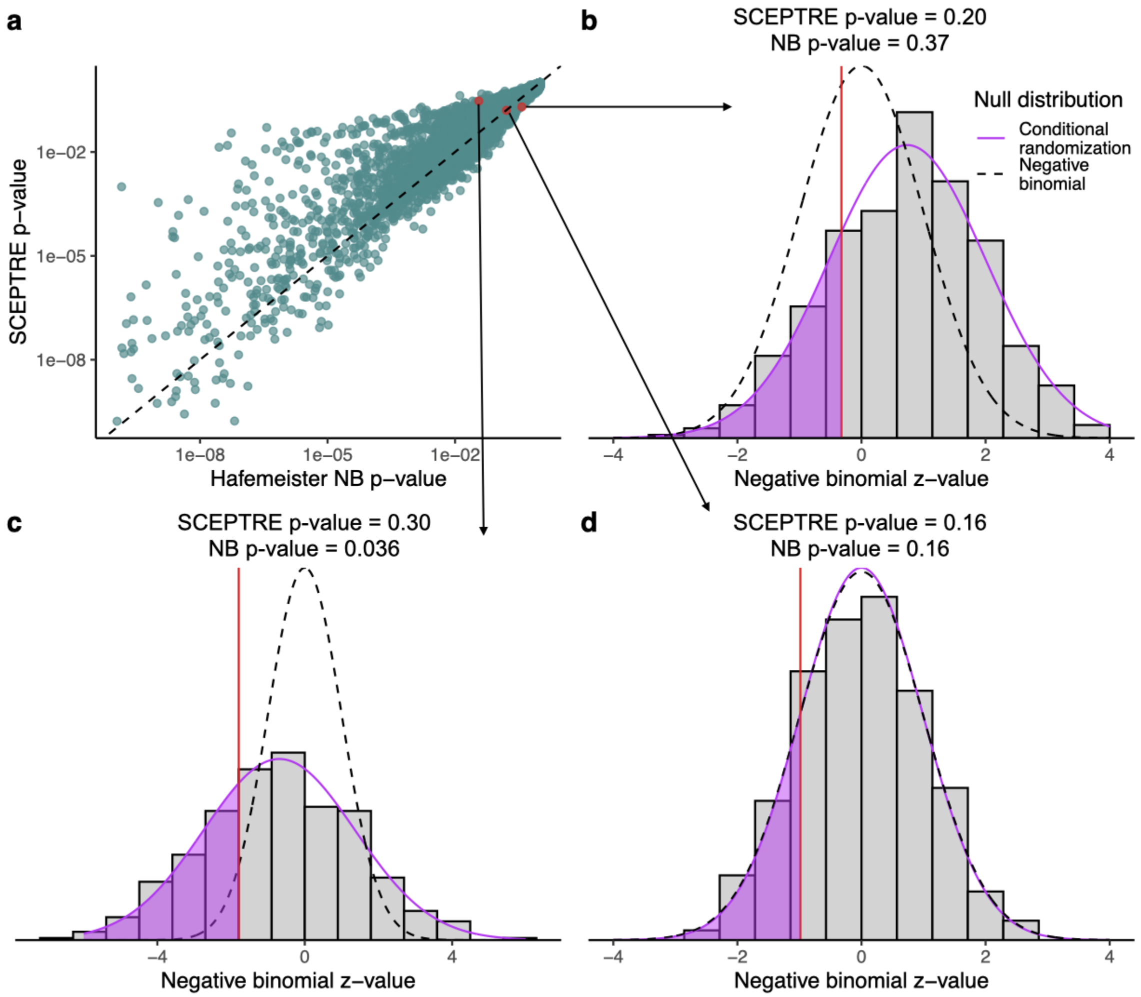

Figure S2: **Comparison of negative binomial and conditional resampling p-values based on the same test statistic. a,** The standard parametric negative binomial p-value versus that obtained from the same test statistic by conditional resampling, for each gene / candidate enhancer pair (both truncated at 10^{-10} for visualization). The two can diverge fairly substantially. **b-d,** Parametric and conditional resampling null distributions for the negative binomial z-value in three cases: the conditional resampling p-value is more significant (b), the parametric p-value is more significant (c), the two p-values are about the same (d).

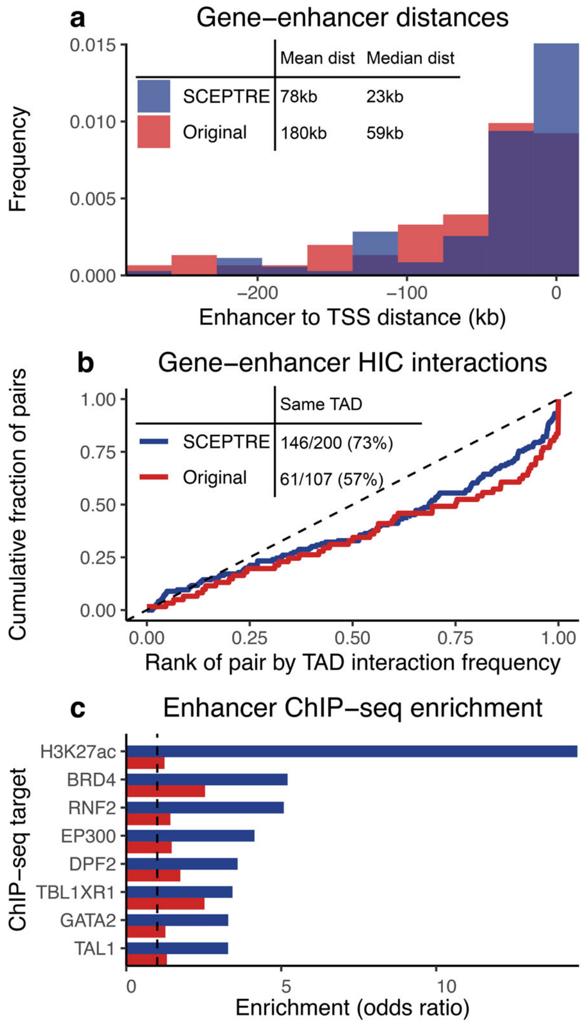

Figure S3: **Discoveries unique to SCEPTRE on the Gasperini data exhibited greater enrichment for biological signal than those unique to the original method.** This figure shows the 200 discoveries unique to SCEPTRE and the 107 discoveries unique to the original method (in contrast to Figure 4, which shows the entire discovery set of both methods). **a,** On average, enhancers discovered by SCEPTRE were less than half the distance to their target genes than those discovered by Gasperini et al. **b,** 73% of the gene-enhancer pairs discovered by SCEPTRE fell within the same TAD, in contrast to 57% of those discovered by the original method. HI-C interaction frequency was similar across methods (though slightly higher for the original), despite the fact that SCEPTRE found 85 more same-TAD pairs. **c,** Enhancers returned by SCEPTRE showed significantly greater enrichment across all ChIP-seq targets, especially H3K27ac.

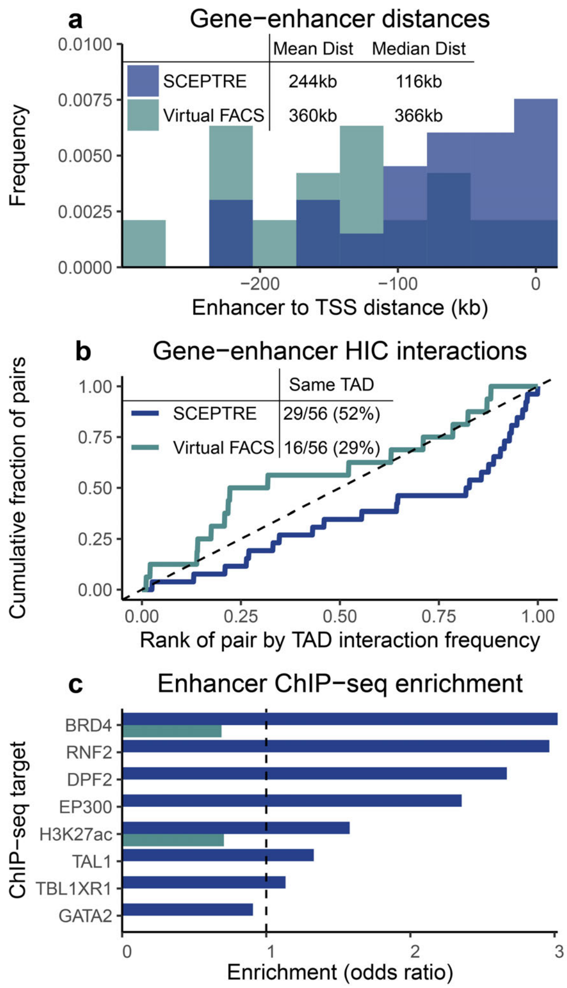

Figure S4: **SCEPTRE-specific discoveries on the Xie et al. data were more enriched for biological signals of regulation than those specific to the original method.** This Figure is analogous to Figure S3 but corresponds to the Xie et al. data; only the discoveries unique to SCEPTRE ($n = 56$) and Virtual FACS ($n = 56$) are depicted. **a,** Gene-enhancer links discovered by SCEPTRE were physically closer (median = 116 kb) to one another than those discovered by Virtual FACS (median = 366 kb). **b,** SCEPTRE pairs exhibited higher HI-C interaction frequency and were more likely to fall within the same TAD (52%) than Virtual FACS pairs (29%). **c,** SCEPTRE pairs showed greater enrichment across all eight cell type-relevant ChIP-seq targets. Virtual FACS odds ratios were exactly equal to zero for six of eight targets.

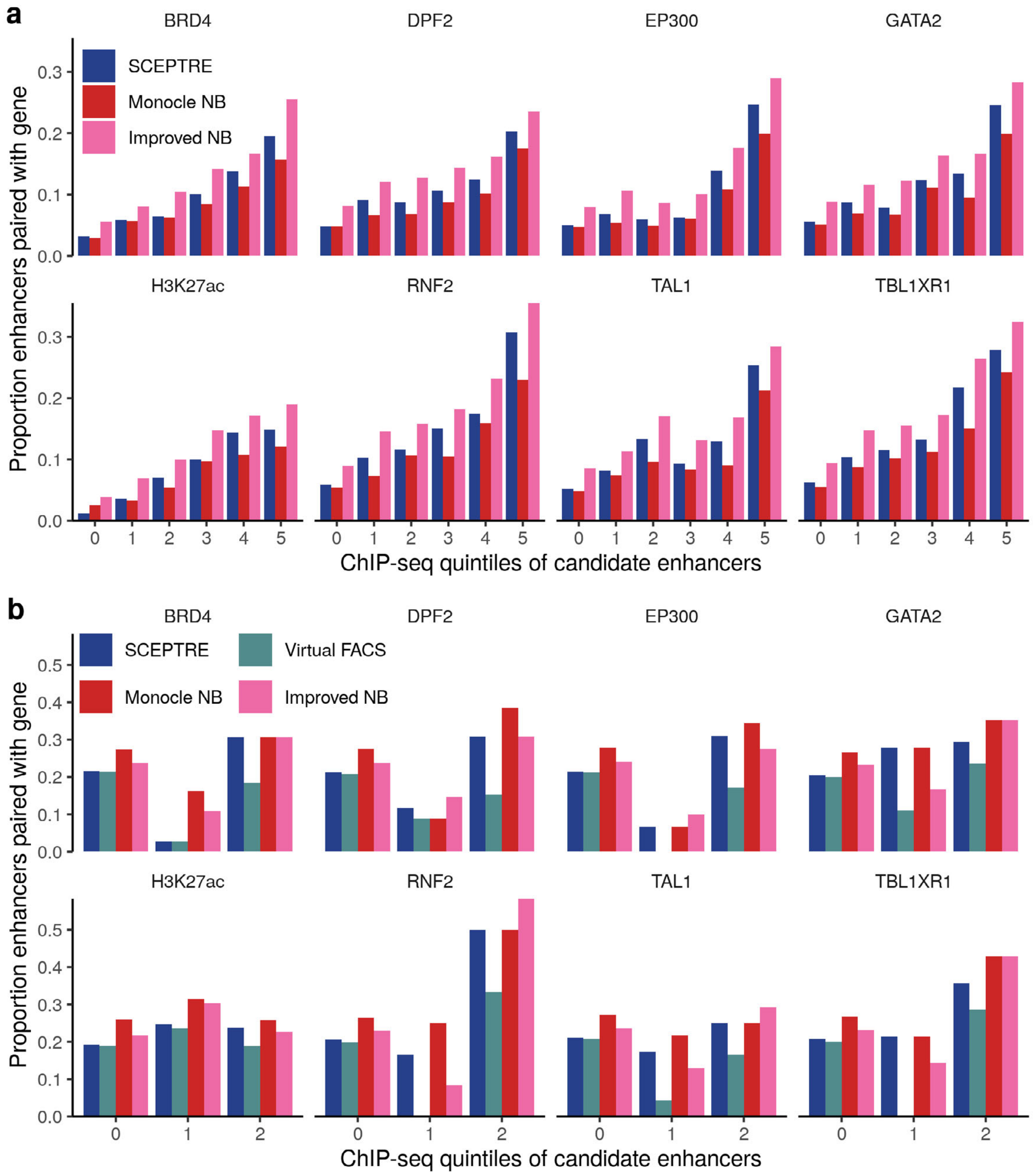

Figure S5: **Details on ChIP-seq enrichment analysis.** Fraction of candidate enhancers linked to gene, broken down by quantile of ChIP-seq signal for (a) Gasperini et al. data (five quantiles used) and (b) Xie et al. data (two quantiles used). "0" indicates that the candidate enhancer did not overlap a ChIP-seq peak. **a**, On the Gasperini et al. data, methods generally paired candidate enhancers in higher ChIP-seq quantiles more frequently. This enrichment was most pronounced for SCEPTRE across all eight ChIP-seq targets. **b**, Trends were less monotonic on the Xie et al. data, possibly due to the fact that Xie et al. used a different strategy for selecting candidate enhancers.

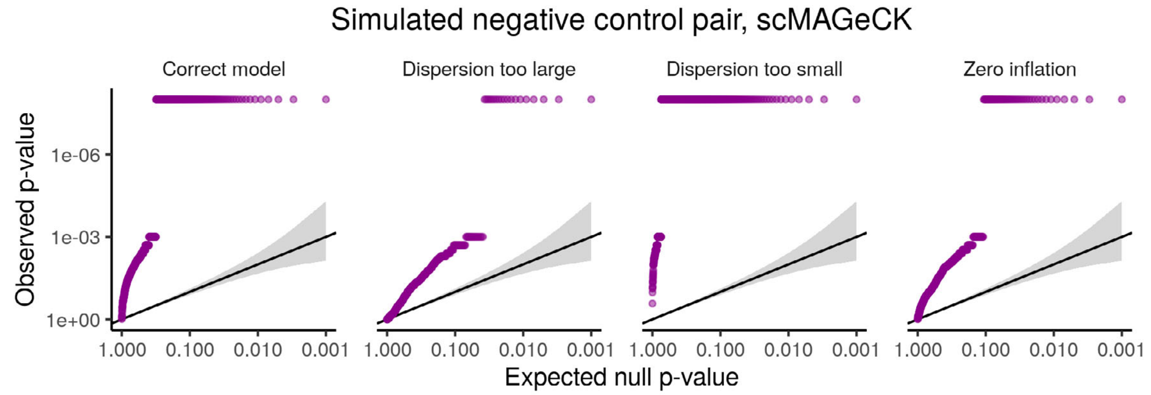

Figure S6: **scMAGeCK simulation study.** Application of an in-house, specialized version of scMAGeCK to the simulated data. Compared to SCEPTRE (Figure 3), scMAGeCK produced inflated p-values.

Quintile	*N* genes	*N* pairs	Mean expression	Percent rejected
1	2108	16253	0.33	0.13%
2	2108	16702	1.5	0.33%
3	2108	16452	3.8	0.62%
4	2108	16692	8.9	0.88%
5	2108	18496	105.0	1.30%

Table S1: To investigate the impact of gene expression level on the sensitivity of SCEPTRE, we binned candidate *cis* genes into five groups based on their mean expression (i.e., mean number of UMIs per cell). Gene-enhancer pairs consisting of genes in higher expression quintiles were more likely to be rejected than pairs consisting of genes in lower expression quintiles, suggesting that SCEPTRE was better able to detect gene-enhancer links for highly-expressed genes. Results reported for Gasperini et al. data.

Tertile	*N* genes	*N* pairs	Mean expression	Percent rejected
1	249	1867	0.001	1.66%
2	249	1868	0.16	3.32%
3	248	1474	68.2	3.12%

Table S2: We replicated the analysis reported in table S1 on the Xie et al. data, binning genes into tertiles rather than quintiles. We observed a similar pattern: genes in the second and third tertile were more likely to be rejected than genes in the first tertile.
